# Predictive risk factors of phenoconversion in idiopathic REM sleep behavior disorder: the Italian study “FARPRESTO”

**DOI:** 10.1007/s10072-022-06374-4

**Published:** 2022-09-10

**Authors:** Monica Puligheddu, Michela Figorilli, Elena Antelmi, Dario Arnaldi, Elisa Casaglia, Ernesto d’Aloja, Luigi Ferini-Strambi, Raffaele Ferri, Gian Luigi Gigli, Francesca Ingravallo, Michelangelo Maestri, Michele Terzaghi, Giuseppe Plazzi, and the FARPRESTO Consortium

**Affiliations:** 1 Sleep Disorders Center, Department of Medical Sciences and Public Health, University of Cagliari , Asse Didattico E., SS 554 Bivio Sestu, Monserrato, 09042 Cagliari, Italy; 2 Department of Neuroscience, Biomedicine and Movement, University of Verona, Verona, Italy; 3 Department of Neuroscience, Rehabilitation, Ophthalmology, Genetics, Maternal and Child Health (DINOGMI), University of Genoa, Largo Daneo 3, 16132 Genoa, Italy; 4 IRCCS Ospedale Policlinico S. Martino, Largo Rosanna Benzi 10, 16132 Genoa, Italy; 5 Department of Medical and Surgical Sciences (DIMEC), University of Bologna, Via Irnerio 49, 40126 Bologna, Italy; 6 Department of Medical Sciences and Public Health, Section of Legal Medicine, University of Cagliari, Cagliari, Italy; 7 Vita-Salute San Raffaele University, Milan, Italy; 8 Department of Clinical Neurosciences, IRCCS San Raffaele Scientific Institute, Milan, Italy; 9 Oasi Research Institute IRCCS, Troina, Italy; 10 Dipartimento di Area Medica (DAME), Università di Udine e Clinica Neurologica e di Neuroriabilitazione, Azienda Sanitaria Universitaria Friuli Centrale, Ospedale “Santa Maria della Misericordia”, Udine, Italy; 11 Department of Clinical and Experimental Medicine, Neurology Unit, University of Pisa, Pisa, Italy; 12 IRCCS Mondino Foundation, Pavia, Italy; 13 Department of Brain and Behavioral Sciences, University of Pavia, Pavia, Italy; 14 IRCCS, Istituto delle Scienze Neurologiche Di Bologna, Bologna, Italy; 15 Department of Biomedical, Metabolic and Neural Sciences, University of Modena and Reggio Emilia, Modena, Italy

**Keywords:** RBD, REM sleep behavior disorder, Neurodegeneration, Parkinson, Biomarkers

## Abstract

Most patients with idiopathic REM sleep behavior disorder (iRBD) will develop an overt α-synucleinopathy over time, with a rate of phenoconversion of 73.5% after 12 years from diagnosis. Several markers of phenoconversion were identified; however, most studies investigated biomarkers separately, with retrospective study designs, in small cohorts or without standardized data collection methods. The risk FActoRs PREdictive of phenoconversion in idiopathic REM sleep behavior disorder: the Italian STudy (FARPRESTO) is a multicentric longitudinal retrospective and prospective study with a cohort of incident (prospective recruitment) and prevalent (retrospective recruitment) iRBD patients, whose primary aim is to stratify the risk of phenoconversion, through the systematic collection by means of electronic case report forms of different biomarkers. Secondary aims are to (1) describe the sociodemographic and clinical characteristics of patients with iRBD; (2) collect longitudinal data about the development of α-synucleinopathies; (3) monitor the impact of iRBD on quality of life and sleep quality; (4) assess the correlation between phenoconversion, cognitive performance, and loss of normal muscle atony during REM sleep; (5) identify RBD phenotypes through evaluating clinical, biological, neurophysiological, neuropsychological, and imaging biomarkers; and (6) validate vPSG criteria for RBD diagnosis. The FARPRESTO study will collect a large and harmonized dataset, assessing the role of different biomarkers providing a unique opportunity for a holistic, multidimensional, and personalized approach to iRBD, with several possible application and impact at different levels, from basic to clinical research, and from prevention to management. The FARPRESTO has been registered at clinicaltrials.gov (NCT05262543).

**Supplementary Information:**

The online version contains supplementary material available at 10.1007/s10072-022-06374-4.

## Background

REM sleep behavior disorder (RBD) is a REM sleep parasomnia first described in 1986 and characterized by the loss of physiological muscle atonia typical of REM sleep and by the presence of abnormal, sometimes violent, motor activity often related to dream content [1]. The observed motor behaviors are often associated to vivid dreams, characterized by an aggressive-defensive content, even if pleasant dreams have been described [2, 3]. The diagnosis of RBD requires video-polysomnographic recording (vPSG), mandatory to identify and quantify the complete or intermittent loss of physiological muscle atonia during REM sleep (REM sleep without atonia, RSWA) and to document any related motor behavior [1]. Recently, the international RBD study group (iRBDSGI) has published comprehensive guidelines for vPSG procedures for diagnosis of RBD [4]. Several manual and automatic methods have been validated to quantify RSWA [5–9]. However, a large study for the validation of RSWA quantification’s method in iRBD population is particularly needed in order to harmonize diagnostic workup of RBD. The exact prevalence of RBD in the general population is not known, but is estimated to range between 0.3 and 1.15%, although this could be underrated [10]. RBD is defined as idiopathic (iRBD) when it is not associated with other obvious neurological or psychiatric disorder [1]. The so-called symptomatic RBD, on the other hand, occurs in association with neurodegenerative diseases belonging to the α-synucleinopathies, narcolepsy, and subacute- or acute-onset conditions involving the central nervous system (structural brain lesions, CNS diseases, drug consumption or alcohol withdrawal, and post-traumatic stress disorder) [1]. In recent years, follow-up studies have shown that most iRBD patients will develop an overt alpha-synucleinopathy over time, with a rate of phenoconversion of 73.5% after 12 years from diagnosis [10–13]. RBD thus represents an early marker of neurodegeneration, a unique open window on the initial phase of alpha-synucleinopathies, encouraging research on neuroprotective therapies [14]. Previous longitudinal studies have indicated older age, presence of hyposmia, abnormal color vision, mild parkinsonism, mild cognitive impairment, autonomic disturbances, nigrostriatal dopaminergic impairment, and severity of loss of RSWA as risk factors for neurodegeneration [10–12, 14–17]. However, most studies investigated biomarkers separately, with retrospective study designs, in small cohorts or without standardized data collection methods across the centers in the case of multicenter studies [18, 19].

To date, there is no reliable candidate biomarkers able to predict phenoconversion, the timing with which this can occur, and the phenotype of the α-synucleinopathy the patient will develop [14, 15]. Furthermore, despite clinical and research evidence suggests that iRBD is a heterogeneous disorder, little attention has been paid to different iRBD phenotypes, and currently, few information are available regarding the impact of iRBD on quality of life. Moreover, large longitudinal cohort studies involving patients with iRBD are scarce.

Identifying different iRBD phenotypes through reliable as well as easily obtainable biomarkers and standardized measures of well-being is crucial to better understand alpha-synucleinopathies, to develop targeted interventions, and to reduce the disease burden. This would significantly increase our understanding on the physiopathological processes of alpha-synucleinopathy since the prodromal phase. Indeed, identifying phenotype clusters with both consolidated and innovative biomarkers may lay the groundwork for a reliable characterization of iRBD patients, likely providing the basis for an efficient stratification of patients to be longitudinally followed.

This article describes the study protocol of the “risk FActoRs PREdictive of phenoconversion in idiopathic REM sleep behavior disorder: the Italian Study” (FARPRESTO), a multicenter Italian observational study with a cohort of incident (prospective recruitment) and prevalent (retrospective recruitment) iRBD patients.

### Aims

The primary aim of the FARPRESTO study is to stratify the risk of phenoconversion, through the systematic collection of different biomarkers (clinical, biological, neurophysiological, neuropsychological, and imaging) in a large cohort of patients with iRBD in Italy.

Secondary aims of the study are (1) to describe the sociodemographic and clinical characteristics of patients diagnosed with iRBD; (2) to collect longitudinal data about the development of alpha-synucleinopathies and estimate of the conversion rate at 3, 5, 7, and 10 years; (3) to monitor over time the impact of iRBD on quality of life and sleep quality by means of validated questionnaires; (4) to assess the correlation between phenoconversion, cognitive performance, and loss of normal muscle atony during REM sleep; (5) to identify RBD phenotypes through valuating clinical, biological, neurophysiological, neuropsychological, and imaging biomarkers; and (6) to validate vPSG criteria for RBD diagnosis. Table 1 summarizes the FARPRESTO aims, while Table 2 condenses all information and biomarkers collected.

**Table 1:** FARPRESTO aims

Aims of the FARPRESTO study	
Primary aim	To identify risk factors of phenoconversion in alpha-synucleinopathies by means of collection of different biomarkers (clinical, biological, neurophysiological, neuropsychological, and imaging biomarkers)
Secondary aims	Describe clinical and sociodemographic characteristics of iRBD To longitudinally assess the development of alpha-synucleinopathy To estimate phenoconversion rate at 3, 5, 7, and 10 years To evaluate quality of life and subjective sleep quality by means of validated questionnaires To assess the correlation between phenoconversion, cognitive performance, and loss of normal muscle atony during REM sleep To identify RBD phenotype through different biomarkers (clinical, biological, neurophysiological, neuropsychological, and imaging biomarkers) To validate vPSG criteria to RBD diagnosis

**Table 2:** FARPRESTO biomarkers

Clinical	Family history Tobacco use Alcohol use Toxic substances exposure Motor symptoms (UPDRS III/MDSUPDRS III) Physical activity Hyposmia Constipation Urgency-urinary dysfunction Erectile dysfunction Orthostatic hypotension Depression Hallucinations EQ5D PSQI
Neurophysiological	RSWA quantification -Visual -Semiautomated
Neuropsychological	Cognitive global assessment (MMSE/MoCA) Neuropsychological evaluation (MCI diagnosis)
Imaging	Neuroimaging (MRI/CT) Spect DAT scan OCT & pupillometry
Laboratory	Skin biopsy Salivary alpha-synuclein Genetic

*(MDS)UPDRS-III*, (Movement Disorders Society) Unified Parkinson’s Disease Rating Scale Part III; *EQ5D*, EuroQol Group’s quality of life questionnaire; *PSQI*, Pittsburg sleep quality index; *RSWA*, REM sleep without atonia; *MMSE*, Mini-Mental State Examination; *MoCA*, Montreal Cognitive Assessment; *MCI*, mild cognitive impairment; *MRI*, magnetic resonance imaging; *CT*, computed tomography; *Spect DAT scan*, single photon emission computed tomography dopamine transporter scan; *OCT*, optical coherence tomography

## Methods/design

### Study design

**Fig. 1:**
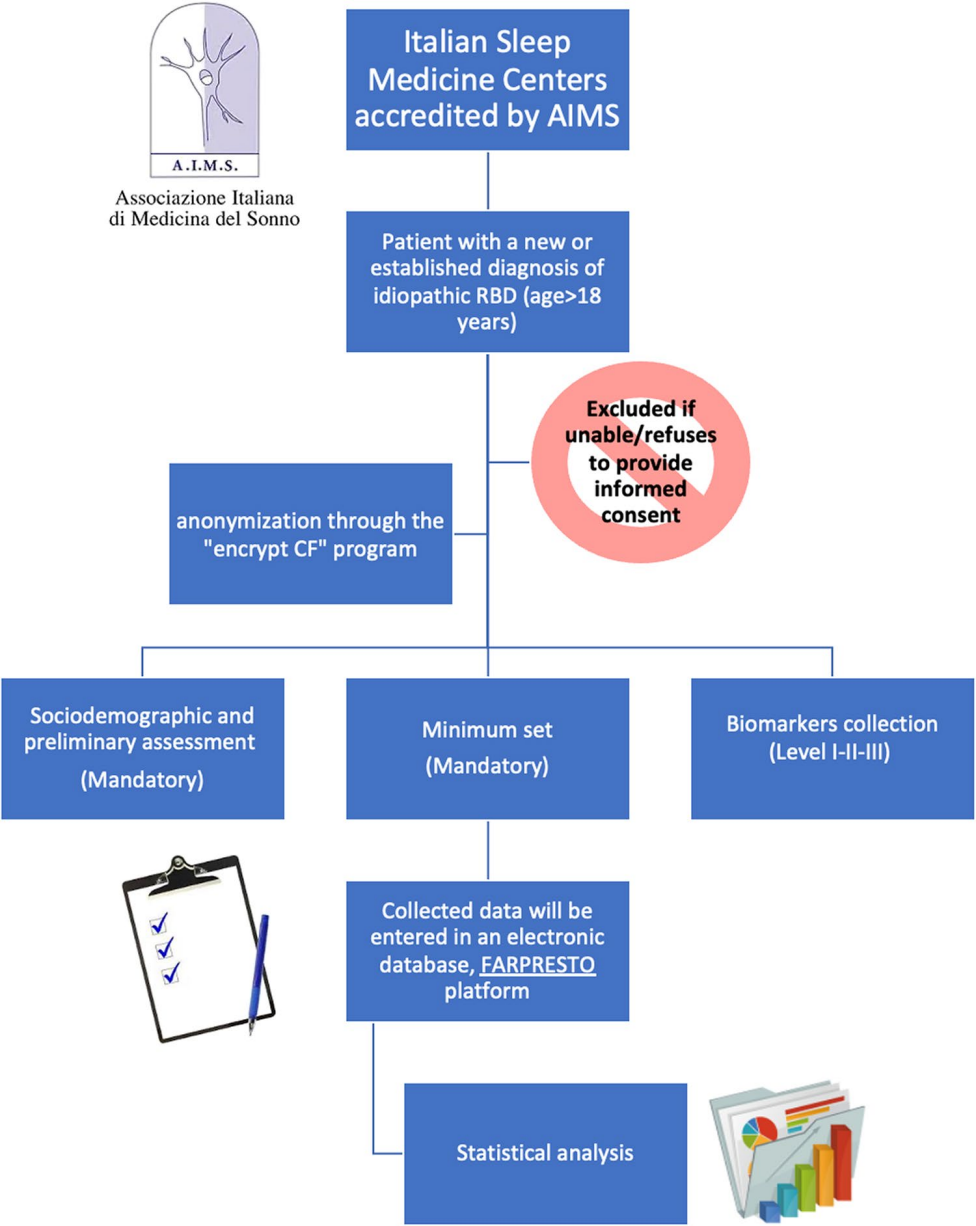
FARPRESTO study design. FARPRESTO study design flowchart (Source clipart: clipart-library.com)

The FARPRESTO study is a national multicentric longitudinal retrospective and prospective study, promoted by the Italian Association of Sleep Medicine (AIMS) and endorsed by the Italian association of patients with RBD (www.sonnomed.it and www.associazionerbd.it). FARPRESTO study was designed to create a large dataset by means of electronic case report forms (e-CRF) collecting sociodemographic and clinical information and data on different biomarkers, namely clinical, biological, neurophysiological, neuropsychological, and imaging biomarkers, retrospectively and prospectively up to 10 years. Figure 1 describes the study design of FARPRESTO.

The study coordinator center is the Sleep Medicine Center of the University of Cagliari, with Principal Investigator M.P. All Italian Sleep Centers accredited by AIMS were invited to participate. At April 2022, 17 centers joined the FARPRESTO Consortium (Table S1).

The scientific committee (SC) will supervise the coordination of the project, data collection, management, analysis and interpretation of data, and writing of the reports of the study. All partners will have access only to data from their center and, upon reasonable request, to the whole anonymized dataset for research purposes.

If, during the study, the opportunity arises to share data with national or international disease registries, a specific amendment to the protocol will be submitted to the ethics committee (EC).

### Study population (inclusion/exclusion criteria, recruitment strategy)

Patients aged 18 years or older, who have been already diagnosed with iRBD, or newly diagnosed iRBD patients, according to the diagnostic criteria of the International Classification of Sleep Disorders second and third edition (ICSD-2, ICSD-3), will be invited to participate during their routine visit. Exclusion criteria are inability to provide consent, to read or write, or to understand the purpose and methods of the study. Patients will be recruited by all Italian Sleep Medicine Centers accredited by AIMS who requested to participate in the Consortium by sending a letter of intent and after approval of the local EC. The recruitment period will vary across the centers according to the EC approval date and will last up to 12 years.

### Study procedures

Retrospective data will be collected from patient records, while the prospective data will be collected during inclusion and follow-up visits, the latter will take place at least once a year. Some data are mandatory for inclusion in the study (i.e., the patient cannot be recruited if these data are not available). Inclusion and follow-up visits comprise a multilevel data collection and assessment with different levels of complexity. In fact, since not all centers collect routinely all types of data, a mandatory minimum set of data entry was established to ensure the quality of the gathered information and the feasibility of stratified analyses. Prospective data will be collected up to 10 years, while no limit has been defined for retrospective data collection as long as mandatory prerequisites are fulfilled. Table 3 summarizes the data collected in the study according to their compulsoriness and the visit in which they are collected.

**Table 3:** FARPRESTO multilevel assessment

Assessments		Mandatory	Inclusion	Follow-up
Sociodemographic	Date and place of birth	X	X	
	Social security number	X	X	
	Sex	X	X	
	Marital status	X	X	
	Years of education		X	
	Employment		X	
Preliminary information	Medical history	X	X	X
	Current therapy	X	X	X
	Tobacco use	X	X	X
	Alcohol use	X	X	X
	Toxic substances exposure	X	X	X
	Family history	X	X	X
	Physical activity	X	X	X
	RBD related injuries		X	X
	Dream content	X	X	X
Minimum set of diagnostic tests	RBD diagnosis (ICSD-2, ICSD-3)	X	X	
	UPDRS III/MDSUPDRS III	X	X	X
	MMSE/MoCA	X	X	X
	Neuroimaging (MRI/CT)	X	X	
Disease outcomes	Phenoconversion			X
	Death			X
I level	RBD onset		X	
	PSG edf		X	
	RSWA quantification		X	
	Hyposmia		X	X
	Constipation		X	X
	Urgency-urinary dysfunction		X	X
	Erectile dysfunction		X	X
	Orthostatic hypotension		X	X
	Depression		X	X
	Hallucinations	X	X	X
	EQ5D		X	X
	PSQI		X	X
II level	Neuropsychological evaluation		X	X
	Spect DAT scan		X	X
III level	Skin biopsy		X	X
	Salivary alpha-synuclein		X	X
	Pupillometry		X	X
	OCT		X	X
	Genetic		X	X

*(MDS)UPDRS-III*, (Movement Disorders Society) Unified Parkinson’s Disease Rating Scale Part III; *MMSE*, Mini-Mental State Examination; *MoCA*, Montreal Cognitive Assessment; *MRI*, magnetic resonance imaging; *CT*, computed tomography; *PSG edf*, polisomnography European Data Format file; *RSWA*, REM sleep without atonia; *EQ5D*, EuroQol Group’s quality of life questionnaire; *PSQI*, Pittsburg sleep quality index; *OCT*, optical coherence tomography

#### Inclusion visit

After signing the informed consent, patients will undergo a multilevel assessment with different degrees of complexity, including sociodemographic, clinical, neurophysiological, neuropsychological, neuroimaging, and laboratory data, with a mandatory minimal dataset for the patient’s recruitment.

During the inclusion visit, sociodemographics and data related to clinical, neuropsychological, and instrumental evaluation will be collected. All the data reported in the inclusion visit should be referred to the time of iRBD diagnosis, even if prior to the enrollment.

Data mandatory for the inclusion are date and place of birth, social security number (securely encrypted), sex, marital status, past and current medical history, current therapy, tobacco and alcohol use, exposure to toxic substances, family history of dementia, movement disorders and sleep disturbances, physical activity, and dream content.

The following sociodemographic and clinical data are not mandatory for inclusion but will be collected whenever they are available: education (years), employment, and self or bedpartner lesions associated to REM sleep-related behaviors (number, type, severity).

Also, the following clinical, neuropsychological, and neuroimaging “minimum set” of diagnostic information are required for the participant’s inclusion: date of RBD diagnosis according to ICSD-2 or ICSD-3 diagnostic criteria; motor assessment by means of the Unified Parkinson’s Disease Rating Scale Part III (UPDRS-III) or Movement Disorders Society-Unified Parkinson’s Disease Rating Scale Part III (MDS-UPDRS-III) indicating date of assessment and global score; global cognitive assessment by the Mini-Mental State Examination (MMSE) or Montreal Cognitive Assessment (MoCA) indicating date of assessment and global score; neuroimaging evaluation (brain MRI or brain CT) specifying date of assessment, report, and Digital Imaging and COmmunications in Medicine (DICOM) file availability. The collection of raw imaging data is not planned at this stage of the study.

The *first level assessment* includes optional clinical-instrumental evaluations, such as date of RBD onset; availability of vPSG European Data Format (EDF) file and hypnogram file in American Standard Code for Information Interchange (ASCII) format for the raw PSG data, RSWA quantification according to a manual scoring method, namely the Montreal and/or the SINBAR methods, and/or to the REM atonia index (RAI) automatic scoring method. Each center may send to the coordinating center the anonymized PSG signals in EDF format and hypnogram files in ASCII format for the quantitative analysis of RSWA, in particular each participating center must provide the characteristics of the signals, regarding their sampling frequency and the presence of the following recorded channels: EEG, EMG (submental, flexor digitorum superficialis, anterior tibialis), ECG, oronasal flow, thoraco-abdominal movements, snoring, oximetry, and body position, by filling in the appropriate file.

Also, the evaluation of non-motor symptoms will be performed either by a semi-structured interview or by validated questionnaires, such as Scales for Outcomes in Parkinson’s Disease-Autonomic Dysfunction (SCOPA-AUT), Hospital Anxiety and Depression Scale (HADS) or Beck Depression Inventory (BDI), presence of hyposmia, constipation, urgency or urinary dysfunction, erectile dysfunction, orthostatic hypotension, depression, and hallucinations, specifying date of assessment, presence, and modality (interview or test). Quality of life and subjective sleep quality will be assessed by means of the EuroQol Group’s quality of life questionnaire (EQ5D) and the Pittsburg sleep quality index (PSQI), respectively.

The *second level evaluation* includes collection of elective data, such as the presence of a comprehensive neuropsychological evaluation, including date of evaluation and the presence or not of mild cognitive impairment, and dopamine transporter (DAT) SPECT availability, date of the exam, the results, and DICOM file availability. The collection of raw imaging data is not planned at this stage of the study. However, a five-pattern visual analysis is requested. In detail, DAT SPECT can be scored as (i) normal, (ii) unilateral putaminal alteration, (iii) bilateral putaminal alteration, (iv) diffuse alteration, and (v) atypical pattern, different from the above.

Finally, the *third level evaluation* comprises non-compulsory laboratory and instrumental data, such as accessibility of the report of the skin biopsy for alpha-synuclein deposit*,* using a 3 mm punch from hairy skin from the leg and from the cervical region. Immunohistochemical analysis of nerve fibers using primary antibodies that simultaneously allow both to unmask the deposits of phosphorylated α-synuclein and to highlight the presence of specific markers of nerve fibers such as the antibody for the pan-neuronal marker. The superposition of both these markers will allow to establish the intraneuronal deposition of phosphorylated α-synuclein deposits; (ii) salivary alpha-synuclein ELISA analysis; (iii) pupillometry obtained through the use of a pupillometer that evaluates the activity of retinal ganglion cells, evaluating the changes in pupil diameter in relation to light stimuli of different intensity, according to the methods previously described [20–22]; optical coherence tomography (OCT), obtained using an instrument (Heidelberg Spectralis) which allows, using reflected light, to reconstruct a three-dimensional image of the thickness of the optic nerve; and (iv) genetic investigation from DNA with a screening panel in which all known gene associated with PD and other synucleinopathies are tested, sampled following a specific protocol.

#### Follow-up visits

During the follow-up visits, that occur at least every 12 months, sociodemographic data will be updated, and results of clinical, neuropsychological, and instrumental evaluations will be collected. At each follow-up visit, assessment of the eventual phenoconversion will be mandatory, specifying date of phenoconversion and diagnosis, choosing among: DLB, PD, or MSA, according to the current diagnostic criteria, or “Other” [23–25]. Parkinsonism was defined as bradykinesia plus at least one of rigidity or rest tremor [24], and dementia was defined as functional impairment in instrumental activities of daily living and with evidence of cognitive impairment on standardized testing [26]. For patients with parkinsonism as the primary disease manifestation, the diagnosis (Parkinson’s disease/multiple system atrophy) will be made according to the treating neurologist. The differential diagnosis incorporates all available follow-up information (i.e., any patient who was initially diagnosed as having Parkinson’s disease at phenoconversion, but who was subsequently found to have multiple system atrophy would be included as affected with multiple system atrophy). The 2017 criteria for probable dementia with Lewy bodies [23] will be used to diagnose the conversion to dementia. In the event of the patient’s death, the cause of death will be collected.

Mandatory first level follow-up evaluation includes neurological examination with the UPDRS-III or MDS-UPDRS-III, global cognitive assessment by means of the MMSE or the MoCA tests (date and report), and assessment of hallucinations. Non-compulsory first level follow-up evaluation comprises assessment of non-motor symptoms, such as hyposmia, constipation, urgency or urinary dysfunction, erectile dysfunction, orthostatic hypotension, depression, and assessment of subjective sleep quality by means of PSQI and quality of life by the EQ5D.

Finally, the second and third level evaluations of the follow-up visit update the same optional instrumental and laboratory examination encompassed in the inclusion visit.

### Statistics

The data will be preliminarily analyzed through descriptive statistics. Phenoconversion at follow-up (primary objective) will be reported as conversion rate (at 1, 3, 5, 7, 10 years), according to survival curve (Kaplan-Meyer method) and also reported for possible conversion predictors. The curves of the individual predictors will be compared with the log-rank test. The independent contribution of the individual predictors will be calculated as a hazard ratio (HR; 95% confidence interval) through a multiple regression analysis (Cox model).

As for the statistical analysis for studying the cut-off values for the identification of RSWA, the cut-off values to differentiate iRBD from healthy controls will be identified by receiver operating characteristic (ROC) analysis, Youden method. The different ROC curves will be compared to each other to search/demonstrate if a given parameter is actually significantly superior to the others. All analyses will be cross-validated. Finally, it will be assessed whether the presence of OSA affects the identified cut-offs, or whether it is necessary to obtain different cut-offs based on the presence/absence of OSA.

Since iRBD is underestimated and clear prevalence data are still lacking, it is arduous to calculate the statistical power of the sample. However, as an example, if we consider that approximately 40% of patients with iRBD show a phenoconversion to parkinsonism and/or dementia within 4 years from diagnosis [27, 28], for a two-sample log-rank test, with a hazard ratio of 0.6, alpha 0.5, and power 0.8, the required total number of patients with iRBD is 303 for n121 phenoconversion events expected.

In addition, if the quantity and quality of the data will permit it, there may be the possibility of analyzing the collected data through more complex methods such as artificial neural network analysis and complex maps.

### Data management

Data will be collected through an ad hoc web-based eCRF available from the “FARPRESTO” platform (www.progettofarpresto.it). The e-CRF will be in Italian. Each participating center will be equipped with a center code and password and each user will have an identification ID and password. Identity of each patient will be anonymized through the “encrypt CF” program starting from social security number, using the SHA3-384 protocol. At the local level, each investigator will only access his own data but can request to the SC to access the whole anonymized dataset for research purposes. After filling in the patient inclusion form, clinicians can access the mandatory “Minimum Set” page for patient recruitment. Subsequently, clinicians will access the optional I, II, and III level assessments. The follow-up form comprises some variables already filled in during the enrollment visit, plus the patient’s conversion status. The field concerning data collected at the inclusion visit can be updated over time to add “historical” data which become known later. The platform e-CRF distinguishes inclusion visit and follow-up visits. The mandatory nature of some fields will be an internal evaluation criterion on the completeness and quality of data collection.

### Ethical approval and informed consent procedures

FARPRESTO will be conducted in accordance with the declaration of Helsinki. The Independent Ethic Committee of the Azienda Ospedaliero Universitaria di Cagliari approved the FARPRESTO study (number protocol PG/2020/5963). Sleep centers submitted the study to their local Ethic Committee for approval. All participants’ personal data will be managed in accordance with the European Union General Data Protection Regulation (GDPR) and national regulation on data protection.

During a follow-up outpatient visit or during scheduled routinary assessments, all patients who meet the inclusion criteria will be provided with information on the purposes and methods of the study. Patients willing to participate in the study will be asked to sign the informed consent form. Patient identification records will be kept confidential and, to the extent permitted by applicable laws and/or regulations, will not be made publicly available. If the results of the study are published, the identity of the participants will remain secret. The signed informed consent form will be kept by the investigators and the patient will receive a copy. Patients will be asked for authorization to contact the general practitioner and/or a contact person in case they do not show up for the control visit, in order to gather information on their state of health.

Each patient will be free to withdraw from the study at any time and to request the destruction of the collected data.

The study has been registered on 02/03/2022 at clinicaltrials.gov with identifier number NCT05262543.

## Discussion

Several prodromal synucleinopathy markers have been proposed as risk factors for neurodegeneration. Among these, the presence of RBD is by far the strongest risk factor carrying the highest positive predictive value for impending synucleinopathy [27]. Thus, patients with iRBD are the ideal target population for testing disease-modifying therapies. However, RBD is a clinical sign likely shared by heterogeneous clinical and neuropathological entities, encompassing mixed populations who eventually develop parkinsonism or dementia first [6, 18, 29].

About 40% of iRBD patients develop parkinsonism and/or dementia within 4 years from diagnosis, but some patients remain still free of neurological disorder after 10 years [28, 30]. The final phenoconversion diagnosis may also be variable, with patients mainly developing PD, DLB, or MSA that, even if they all belong to alpha-synucleinopathies, have different clinical characteristics and prognosis. From a clinical point of view, iRBD patients also show heterogeneity, not showing all patients the same alterations, rather presenting a puzzle of different associations between early markers with only some of them showing hyposmia, cognitive impairment, and autonomic disorders [11]. Moreover, some but not all iRBD patients show neuroimaging alterations, including cortical and subcortical magnetic resonance imaging (MRI) abnormalities and single photon emission tomography (SPECT) nigro-striatal dopaminergic impairment [18]. All these biomarkers have been considered to be predictors of future phenoconversion in iRBD patients [11, 18, 31].

The FARPRESTO study will permit to comprehensively characterize a large cohort of patients with iRBD, assessing clinical, biological, neurophysiological, neuropsychological, and imaging biomarkers through a 10-year follow-up period. Besides, our multilevel data collection approach, with a mandatory minimum set of data entry that guarantees the quality of the gathered information, makes the study more inclusive while not losing the opportunity to collect data usually available only at selected centers. Indeed, the use of routine data not collected for a specific project may lead to a low quality relatively to their accuracy, precision, and completeness. Thus, the nature of data (namely, if they were routinely collected or ad hoc collected for the FARPRESTO study) will always be reported in the study publications.

The longitudinal analysis will allow to evaluate the development of neurodegenerative pathologies of the spectrum of alpha-synucleinopathies, such as PD, DLB, and MSA, and to estimate the conversion rate at 3, 5, 7, and 10 years, exploring the baseline risk factors of phenoconversion. Moreover, the longitudinal design is a unique opportunity for describing different disease progression trajectories and to investigate the factors associated with different outcomes.

The FARPRESTO will allow to better phenotype and stratify the iRBD population helping to advance our understanding on the pathophysiological processes of alpha-synucleinopathy from the prodromal phase and to better distinguish different risk profiles of neurodegeneration. Thus, there is a huge need for identifying the pool of more sensitive, specific, and cost affordable biomarkers, capable of predicting phenoconversion to the full-blown neurodegenerative disease, in terms of timing and type of phenoconversion. This information will be essential for clinicians, patients, and family and will permit to better identify the ideal population for neuroprotective and disease-modifying trials. In fact, several disease-modifying therapies are now in development, including but not limited to monoclonal antibodies against alpha-synucleinopathy (ClinicalTrials.gov identifiers: NCT04534023, NCT05109364, NCT04152655, NCT04739423). Patients with prodromal synucleinopathy, such as those with iRBD, are the ideal target to test disease-modifying therapies because the neurodegeneration is still in an early stage and the likelihood to rescue both brain structure and function is higher; thus, even if this is not an explicit aim of the study, FARPRESTO could also provide a trial-ready cohort of iRBD patients.

Moreover, this study will prospectively collect information regarding patients’ quality of life, mood, and alterations of specific cognitive domains. This is important not only for clinicians and researchers but also for patients and family who need to know what they should expect will happen over time. This information, along with neurophysiological, imaging, and biological data and phenoconversion timing, will provide the opportunity to better characterize different clinical iRBD phenotypes. In particular, it will be interesting to analyze the correlation between phenoconversion, cognitive performance, and the degree of loss of normal muscle atonia during REM sleep, as these assessments can be performed with reasonable ease in all centers. The combined and holistic evaluation of qualitative and quantitative information will also allow an increasingly tailored and implemented approach to patient care. Furthermore, this study will allow to validate vPSG criteria, in particular RSWA quantification methods, in a large cohort of iRBD patients, in order to harmonize RBD diagnostic workup. In addition, the collected data could be analyzed with highly complex statistical methods (e.g., artificial neural network and complex maps) which may allow stratification of iRBD subjects into subtypes in order to identify the most significant clusters of association for the purpose of predicting phenoconversion. Through neural network analysis approaches, it is now possible to find out complex correlations between data from different sources (i.e., clinical examinations, questionnaires, biological data, and imaging and neurophysiological techniques) and to identify sub-groups of patients sharing the same cluster of substantial characteristics. The same technique would also allow identifying the importance that certain types of information have in relation to each particular phenotype. Identifying different iRBD phenotypes through established as well as innovative biomarkers and standardized measures of well-being is crucial to better understand alpha-synucleinopathies, to develop targeted interventions, and to reduce the disease burden.

Furthermore, the creation of an organized data collection system, currently at national level, could favor future international cooperation. The implementation of data relating to populations with different genetic and environmental backgrounds could not only increase the sample and statistical power but also favor an increasingly uniform standard of care and patient management.

In conclusion, FARPRESTO provides a unique opportunity for a holistic, multidimensional, and personalized approach to iRBD, with several possible application and impact at different levels, from basic to clinical research, and from prevention to management. The possible applications and impacts of the project may be summarized as the following: (a) better understanding of iRBD and alpha-synucleinopathies; (b) developing a personalized approach to iRBD patients; and (c) favoring targeted interventions to reduce the disease burden, especially through personalized prevention strategies.

## Supplementary Information

Below is the link to the electronic supplementary material.

Supplementary file1 (PDF 71 KB)

## Abbreviations

*AIMS*: Italian Association of Sleep Medicine
*ASCII*: American Standard Code for Information Interchange
*BDI*: Beck Depression Inventory
*CT*: Computed tomography
*DICOM*: Digital Imaging and Communications in Medicine
*DLB*: Dementia with Lewy bodies
*EC*: Ethics committee
*e-CRF*: Electronic case report forms
*EDF*: European Data Format
*ELISA*: Enzyme linked immunosorbent assay
*EQ5D*: EuroQol Group’s quality of life questionnaire
*HADS*: Hospital Anxiety and Depression Scale
*MCI*: Mild cognitive impairment
*MDS-UPDRS-III*: Movement Disorders Society-Unified Parkinson’s Disease Rating Scale Part III
*MMSE*: Mini-Mental State Examination
*MoCA*: Montreal Cognitive Assessment
*MRI*: Magnetic resonance imaging
*MSA*: Multiple system atrophy
*OCT*: Optical coherence tomography
*PD*: Parkinson’s disease
*PGP*: Protein gene product
*PSQI*: Pittsburg sleep quality index
*RAI*: REM sleep atonia index
*(i)RBD*: (Idiopathic) REM sleep behavior disorder
*RSWA*: REM sleep without atonia
*SCOPA-AUT*: Scales for Outcomes in Parkinson’s Disease-Autonomic Dysfunction
*UPDRS-III*: Unified Parkinson’s Disease Rating Scale Part III
*vPSG*: Video-polysomnography
