# Correction to: Predictive risk factors of phenoconversion in idiopathic REM sleep behavior disorder: the Italian study “FARPRESTO”

**DOI:** 10.1007/s10072-022-06492-z

**Published:** 2022-11-07

**Authors:** Monica Puligheddu, Michela Figorilli, Elena Antelmi, Dario Arnaldi, Elisa Casaglia, Ernesto d’Aloja, Luigi Ferini-Strambi, Raffaele Ferri, Gian Luigi Gigli, Francesca Ingravallo, Michelangelo Maestri, Michele Terzaghi, Giuseppe Plazzi, the FARPRESTO Consortium

**Affiliations:** 1 Sleep Disorders Center, Department of Medical Sciences and Public Health, University of Cagliari, Asse Didattico E., SS 554 Bivio Sestu, Monserrato, 09042 Cagliari, Italy; 2 Department of Neuroscience, Biomedicine and Movement, University of Verona, Verona, Italy; 3 Department of Neuroscience, Rehabilitation, Ophthalmology, Genetics, Maternal and Child Health (DINOGMI), University of Genoa, Largo Daneo 3, 16132 Genoa, Italy; 4 IRCCS Ospedale Policlinico S. Martino, Largo Rosanna Benzi 10, 16132 Genoa, Italy; 5 Department of Medical Sciences and Public Health, Section of Legal Medicine, University of Cagliari, Cagliari, Italy; 6 Vita-Salute San Raffaele University, Milan, Italy; 7 Department of Clinical Neurosciences, IRCCS San Raffaele Scientific Institute, Milan, Italy; 8 Oasi Research Institute IRCCS, Troina, Italy; 9 Dipartimento di Area Medica (DAME), Università di Udine e Clinica Neurologica e di Neuroriabilitazione, Azienda Sanitaria Universitaria Friuli Centrale, Ospedale “Santa Maria della Misericordia”, Udine, Italy; 10 Department of Medical and Surgical Sciences (DIMEC), University of Bologna, Via Irnerio 49, 40126 Bologna, Italy; 11 Department of Clinical and Experimental Medicine, Neurology Unit, University of Pisa, Pisa, Italy; 12 IRCCS Mondino Foundation, Pavia, Italy; 13 Department of Brain and Behavioral Sciences, University of Pavia, Pavia, Italy; 14 IRCCS, Istituto delle Scienze Neurologiche Di Bologna, Bologna, Italy; 15 Department of Biomedical, Metabolic and Neural Sciences, University of Modena and Reggio Emilia, Modena, Italy

**Correction to: Neurological Sciences (2022)**

10.1007/s10072-022-06374-4

Originally, the article was published with an error. The author Dario Arnaldi should only have 2 affiliations, thus, affiliation 5 should be removed from his address.

The original article has been corrected.

