# Supplementary material for: Predictive risk factors of phenoconversion in idiopathic REM sleep behavior disorder: the Italian study “FARPRESTO”
Source: Neurol Sci. 2022 Sep 10;43(12):6919–28. doi: 10.1007/s10072-022-06374-4 (PMC9663351; doi:10.1007/s10072-022-06374-4)
Supplement: Supplementary file 1 — Supplementary file1 (PDF 71 KB) [file 10072_2022_6374_MOESM1_ESM.pdf]

Table S1. The FARPRESTO Consortium

1.Monica Puligheddu, Michela Figorilli, Elisa Casaglia<br>ID Sleep Disorder Centre, University of Cagliari
2. Claudio Liguori,[a-b] Fabio Placidi,[a-b] Francesca Izzi,[a] Nicola Biagio Mercuri,[a-b-c]<br>a Sleep Medicine Center, Neurology Unit, University Hospital of Rome Tor Vergata<br>b Department of Systems Medicine, University of Rome Tor Vergata<br>c Santa Lucia Foundation, Rome
3. Dario Arnaldi,[a,b] Pietro Mattioli,[a] Flavio Nobili[a,b]<br>a Clinical Neurology, University of Genoa<br>b IRCCS Ospedale Policlinico San Martino, Genoa
4. Federica Provini,[a,b] Luca Baldelli,[a] Pietro Cortelli[a,b]<br>a Department of Biomedical and NeuroMotor Sciences (DiBiNeM), University of Bologna, Bologna, Italy<br>b IRCCS Istituto delle Scienze Neurologiche di Bologna, Bologna, Italy
5. Michele Terzaghi,[a,b], Giuseppe Fiamingo[b]<br>a Mondino Foundation IRCCS, Pavia, Italy<br>b Department of Brain and Behavioral Sciences, University of Pavia, Pavia, Italy.
6. Valerio Brunetti[a,b], Giacomo Della Marca[a,b], Paolo Calabresi[a,b]<br>a Dipartimento di Neuroscienze, Università Cattolica del Sacro Cuore, Rome, Italy<br>b Fondazione Policlinico Universitario Agostino Gemelli IRCCS, Rome, Italy
7. Luigi Ferini Strambi [a,b], Marco Zucconi [b], Alessandro Oldani [b], Sara Marelli [b], Alessandra Castelnuovo [b], Francesca Marta Casoni [b]<br>a Vita-Salute San Raffaele University, Milan, Italy<br>b IRCCS San Raffaele Scientific Institute, Department of Clinical Neurosciences, Neurology-Sleep Disorders Centre, Milan, Italy
8. Enrica Bonanni, Michelangelo Maestri, Gabriele Siciliano, Domeniko Hoxhaj, Alessia Pascazio, Roberto Ceravolo.<br>UO Neurologia, Dipartimento di Medicina Clinica e Sperimentale, Università di Pisa
9. Biancamaria Guarnieri, Ilde Pieroni, Lucia velluto, Gianluigi Cerroni<br>Centro di medicina del sonno AIMS, UO di Neurologia, Casa di cura privata accreditata Villa Serena, Città S. Angelo, Pescara
10. Raffaele Ferri<br>Centro di medicina del sonno AIMS, IRCCS Oasi Maria SS, Troina

11. Andrea Gagliardo,<br>U.O. Neurofisiopatologia Laboratorio Del Sonno Clinical Course. Palermo
12. Mariantonietta Savarese<br>Centro di Medicina del Sonno (Multidisciplinare),U.O. Neurologia Universitaria "FrancoMichele Puca", Azienda Ospedaliero Universitaria Consorziale Policlinico di Bari
13. Riccardo Cremascoli<br>Centro di Medicina del sonno IRCCS Auxologico Piancavallo, Oggebbio (VB)
14. Elisa Testani<br>Neurologia e Neurofisiologia Clinica,AOU Senese
15. Fabio Placidi<br>Centro del sonno, UNIT trattamento neurologico della fragilità. UOC Neurologia, Policlinico TorVergata, Roma
16. Gianluca Rossato<br>U.0 . Neurologia IRCCS Sacro Cuore Don Calabria (Negrar, VR).
17. Gian Luigi Gigli, Gaia Pellitteri, Sara Tartaglia, Giovanni Ermanis<br>Azienda Sanitaria Universitaria Friuli Centrale (ASU FC) - SOC Clinica Neurologica e di Neuroriabilitazione Udine
